# Trial to Incentivise Adherence for Diabetes (TRIAD): study protocol for a randomised controlled trial

**DOI:** 10.1186/s13063-017-2288-6

**Published:** 2017-11-17

**Authors:** Marcel Bilger, Mitesh Shah, Ngiap Chuan Tan, Kaye Louise Howard, Hui Yan Xu, Ecosse Luc Lamoureux, Eric Andrew Finkelstein

**Affiliations:** 1 Health Services and Systems Research, Duke-NUS Medical School, 8 College Road, 169857 Singapore, Singapore; 2 SingHealth Polyclinics, Singapore, Singapore; 3 Singapore Eye Research Institute, Singapore National Eye Centre, Singapore, Singapore; 4 Duke Global Health Institute, Duke University, Durham, NC USA

**Keywords:** Diabetes, Medication adherence, Blood glucose monitoring, Physical activity, Financial incentive, Behaviour change

## Abstract

**Background:**

Many people with diabetes have suboptimal glycaemic control due to not being adherent to their treatment regimen. Behavioural economic theory suggests that the lack of adherence results from the disconnect between the timing of when costs and benefits accrue. One strategy to address this discontinuity is to offer patients a near-term benefit, such as a financial reward. Whereas there is evidence that rewards can improve treatment adherence and sometimes health outcomes, further research is needed to determine whether rewards are more effective when targeting processes or intermediary health outcomes. In the Trial to Incentivise Adherence for Diabetes (TRIAD) we test whether adding financial incentives to usual care can improve HbA1c levels among people with diabetes and whether the financial incentives work better when targeting processes (adherence to blood glucose testing, medication, and daily physical activity) or the primary intermediary health outcome of self-monitored blood glucose within an acceptable range.

**Methods/design:**

TRIAD is a randomised, controlled, open-label, single-centre superiority trial with three parallel arms. A total of 240 patients with suboptimally controlled diabetes (HbA1c ≥ 8%) from a polyclinic in Singapore are block-randomised (blocking factor: current vs. new glucometer users) into three arms, namely (1) usual care (UC) only, (2) UC with process incentive and (3) UC with outcome incentive, in a 2:3:3 ratio. Masking the arm allocation will be precluded by the behavioural nature of the intervention but blocking size will not be disclosed to protect concealment. The primary outcome (change in HbA1c level at month 6) will be measured by a laboratory that is independent from the study team. Secondary outcomes (at month 6) include the number of blood glucose testing days, glucose readings within the normal range (between 4 to 7 mmol/L), medication-adherent days, physically active days, and average incentives earned and time spent administrating the incentives.

**Discussion:**

This study will provide evidence on whether financial incentives can cost-effectively improve glycaemic control. It will also provide evidence on the benefit incidence of interventions involving financial incentives. By comparing process to outcome incentives, this study will inform the design of future incentive strategies in chronic disease management and beyond.

**Trial registration:**

ClinicalTrials.gov registry, ID: NCT02224417. Registered on 22 August 2014.

**Electronic supplementary material:**

The online version of this article (doi:10.1186/s13063-017-2288-6) contains supplementary material, which is available to authorized users.

## Background

### Rationale

In 2014, there were 422 million adults with diabetes worldwide [1]. Diabetes is associated with a host of adverse complications, including myocardial infarction, stroke, blindness, kidney failure and severe neuropathy that may result in amputations [2]. The global direct healthcare cost of diabetes and its complications was estimated to exceed USD827 billion in 2014 [3, 4]. In Singapore, diabetes is the cause of 10.4% of all disability-adjusted life years (DALYs) lost, which represents the fourth most important disease burden [5].

Diabetes management primarily consists of promoting glycaemic control via diet, physical activity; and, if necessary, medication. Medication generally includes oral hypoglycaemic agents, which may be followed by injectable insulin when blood glucose remains suboptimally controlled. In 2012, higher-than-optimal blood glucose was responsible for 2.2 million of the 3.7 million deaths caused by diabetes worldwide [2]. In 2010 in Singapore, about a third (32%) of known people with diabetes had suboptimal glycaemic control as defined by a concentration of glycated haemoglobin (HbA1c) of 8.0% or more [6].

While treatment effectiveness crucially depends on the quality of care provided by health professionals (e.g. efficacious medications, clear and appropriate advice, relevant health education and support), patient engagement is especially important to chronic disease management as most of the treatment takes place outside the healthcare system. In 2003, the World Health Organisation reported that, worldwide, only 50% of patients suffering from chronic diseases were adherent to their treatment. Regarding diabetes, all aspects of treatment (glucose monitoring, administration of medication, diet and physical activity) were affected by non-adherence. As a result, much research was conducted in efforts to improve diabetes management. Meta-evidence on the average reduction in HbA1c levels achieved by various types of interventions shows that education on diabetes self-management reduces HbA1c levels by 0.57 percentage points [7], self-monitoring of glucose levels reduces these levels by 0.25 percentage points [8], behavioural interventions targeting physical activity reduces HbA1c levels by 0.32 percentage points [9], and computer-based interventions to improve diabetes self-management reduces HbA1c levels by 0.20 percentage points [10]. In their review of quality improvement strategies on the management of diabetes, Tricco and colleagues found an average reduction in HbA1c levels of 0.37 percentage points and note that strategies targeting patients were on average more effective [11]. While all were statistically significant, the above meta-estimates reveal modest-to-moderate effect sizes. Improvement in glycaemic control in the population remains extremely slow [12], and more generally, the rise in number of people with multiple comorbidities makes treatment adherence even more difficult to achieve overall.

Behavioural economic theory may provide an explanation for the lack of adherence to treatment recommendations: the benefits of greater efforts of adherence only arise well into the future whereas the costs are immediate. When costs and benefits are so far apart, individuals have been shown to be biased towards the present, which results in decisions that are time-inconsistent and that generate significant regret [13]. One strategy that addresses such present bias is to provide patients with near-term benefits. This can be achieved by providing patients with financial incentives that are contingent on treatment adherence. Recent research on financial incentives uses behavioural economics in efforts to generate the largest behavioural change. For instance, studies are leveraging patients’ loss aversion with deposit contract incentives [14] and adherence contingent rebates [15], and patients’ probabilistic assessment bias with lottery incentives [14, 16, 17].

Evidence on the effectiveness of financial incentives for diabetes management is scarce. Long and colleagues found a reduction in HbA1c level of 0.45 percentage points among African American Veterans with diabetes by tying rewards to HbA1c reductions but this effect was not statistically significant [18]. Sen and colleagues found that a lottery incentive tied to glucose monitoring improved monitoring rates by 19 to 23 percentage points among uncontrolled people with diabetes in a US primary-care medical home practice [19].

The first primary objective of this trial is to determine whether adding financial incentives to usual care (UC) can improve HbA1c levels among people with suboptimally controlled type-2 diabetes in the primary-care setting. The second primary objective is to determine whether financial incentives should be directed at processes (adherence to blood glucose testing, medication, and daily physical activity) or at achieving intermediary health outcomes (self-monitored blood glucose within acceptable range). This trial will provide novel head-to-head evidence on which strategy works best. In addition, the trial will assess the effect of financial incentives on treatment adherence and whether such incentives constitute cost-effective intervention strategies for diabetes management. Finally, explanatory analysis will aim at determining whether patient perception about diabetes management is altered by the interventions, uncovering factors that might moderate the effect of financial incentives, and identifying socioeconomic groups that may benefit more from the interventions.

### Objectives

#### Primary objective 1

To determine whether complementing UC with financial incentives for good treatment adherence is superior to UC alone in reducing HbA1c at 6 months.

#### Primary objective 2

To determine whether incentivising intermediate health outcomes (self-monitored blood glucose levels within acceptable range) is superior to incentivising intermediate processes (blood glucose testing, physical activity, and medication adherence) in reducing HbA1c at 6 months.

#### Secondary objective 1

To assess whether complementing UC with financial incentives for good treatment adherence is superior to UC alone in improving treatment adherence as measured by the proportion of glucose tests and medications taken as prescribed and average number of daily steps taken between baseline and 6 months.

#### Secondary objective 2

To assess whether incentivising health outcomes is superior to incentivising intermediate processes in improving treatment adherence as measured by the proportion of glucose tests and medications taken as prescribed and average number of daily steps between taken baseline and 6 months.

#### Secondary objective 3

To assess which intervention (i.e. incentivising health outcomes or processes) has the most favourable incremental cost-effectiveness ratio (ICER) for HbA1c reductions between baseline and 6 months compared to UC alone.

## Methods/design

### Trial design

**Fig. 1:**
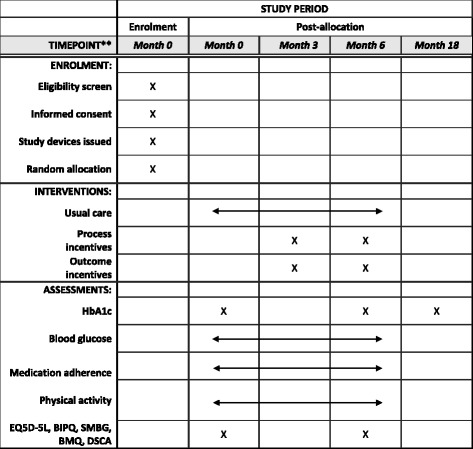
Standard Protocol Items: Recommendations for Interventional Trials (SPIRIT) Figure. *HbA1c* glycated haemoglobin, *EQ-5D-5L* European Quality of Life-5 Dimensions-5 Levels, *BIPQ* Brief Illness Perception Questionnaire, *SMBG* self-monitoring of blood glucose, *BMQ* Beliefs about Medication Questionnaire, *DSCA* Diabetes Self-Care Activities

The Trial to Incentivise Adherence for Diabetes (TRIAD) is designed as a randomised, controlled, single-centre superiority trial with three parallel arms. A total of 240 people with suboptimally controlled diabetes will be block-randomised (blocking factor: current vs. new glucometer users) into the usual care (UC), process incentive and outcome incentive arms in a 2:3:3 ratio. The study intervention will last for 6 months. The primary outcome is change in HbA1c level as measured by blood tests at baseline and month 6. This protocol conforms to the Standard Protocol Items: Recommendations for Interventional Trials (SPIRIT) guidelines. (Additional file 1: SPIRIT Checklist and Fig. 1: SPIRIT Figure).

### Study setting and eligibility criteria

All the participants with suboptimally controlled diabetes will be recruited among the patients from the SingHealth Polyclinic in Geylang, Singapore. Singapore is a densely populated island city-state that has a population of 5.5 million. It is made up of Chinese (76.2%), Malay (15%), Indian (7.4%) and other (1.4%) ethnicities. It is a multi-cultural country with the most widely spoken languages being English, Mandarin, Malay and Tamil. A large proportion of those who speak Malay and Tamil are also proficient in English.

SingHealth Polyclinics are part of SingHealth, which is Singapore’s largest public healthcare group. SingHealth has a network of nine Polyclinics in Singapore, providing primary healthcare to local communities that is designed to be affordable and accessible to all. Services provided at the polyclinics include medical care for acute and chronic conditions, medical examinations and screening, minor surgical procedures and clinical laboratory services. SingHealth Polyclinics has a research department with three signature research programmes, including Chronic Disease Management, Education Research and Innovation in Primary Care. The study will be conducted in collaboration with Duke-NUS Medical School as part of the SingHealth Duke-NUS Academic Medical Centre.

#### Inclusion criteria

- Have suboptimally controlled diabetes at baseline. Suboptimally controlled diabetes is defined by a HbA1c level of 8.0% or greater. As such, patients will be required to have at least one of two HbA1c readings of 8.0% or greater in the past 6 months
- Be prescribed at least one oral diabetic medication for at least 3 months
- Aged between 21 and 70 years of age
- Be Singaporean citizens or permanent residents
- Be able to converse in English or Mandarin

#### Exclusion criteria

- Patients taking injectable insulin therapy
- Patients with significant comorbid conditions such that they are unlikely to be able to take their medications without assistance from a third party
- Patients who are pregnant
- Patients who answer ‘yes’ to at least one question of the Physical Condition Questionnaire, or indicate having a heart condition or disease, a stroke, or a lung disease (chronic obstructive pulmonary disease or asthma), or report that an immediate family member (mother, father, sister or brother) had a myocardial infarction or died of a heart-related disorder before age 55 (men) or 65 (women) years will be excluded from the study unless they receive approval from a physician who is not part of the study team

### Participant timeline and study arms

**Fig. 2:**
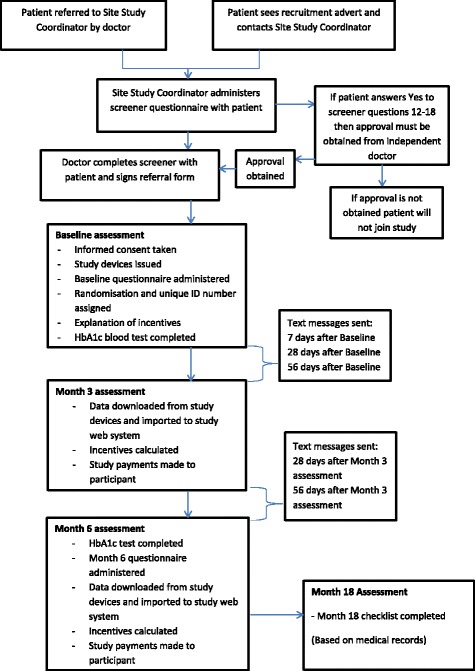
Trial to Incentivise Adherence for Diabetes (TRIAD) participant timeline

Participants in all three study arms will be issued with one Fitbit Zip™ and one eCAP™. A Fitbit Zip™ (Fitbit Inc., San Francisco, CA, USA) is a pedometer with wireless synchronisation to smartphones and computers. It tracks daily steps taken, calories burned and distance walked. An eCAP™ (Information Mediary Corporation, Ottawa, ON, Canada) is a medication-event monitoring system with an inbuilt electronic tag which records the time whenever it is closed. Participants will also use a glucometer for the study. A glucometer is a medical device that measures blood glucose levels. It is estimated that approximately half of the study participants will already use a glucometer before joining the study. Participants without a glucometer will be provided with a Nipro TRUEresult™ glucometer which they may keep after the study ends. The project coordinator will label the study devices with the participant’s unique ID number as allocated at randomisation. The clinical research coordinator (CRC) will demonstrate how to adequately use all study devices. Participants who have difficulties with their glucometer will be referred to a health counsellor at the polyclinic. Participants in all study arms will attend baseline, month 3 and month 6 assessments. At the baseline and month-6 assessments, the participants will fill out survey questionnaires (see the ‘Data collection’ section below) and take HbA1c blood tests. At the month-3 and month-6 assessments, the CRC will use the data from the three study devices to evaluate treatment adherence and the corresponding incentive payments in supermarket vouchers when applicable. At the month-18 assessment, a month-18 checklist based on participants’ medical records will be filled up by the CRC. Participants’ HbA1c blood test result since their month-6 assessment and any change in diabetes medication regimen (e.g. initiation of insulin) will be recorded. Figure 2 displays the timeline of the participants in the study.

Participants in the UC arm will not receive any financial incentives for meeting the recommended goals, but they will receive a non-contingent payment at the end of the intervention. Participants in the process incentive arm will have the opportunity to earn financial incentives for meeting specified goals for physical activity, medication adherence and blood glucose testing. The participants in the outcome incentive arm will have the opportunity to earn financial incentives for meeting specified goals for blood glucose testing. In addition, participants in all study arms will be compensated to attend the assessment visits. Table 1 summarises the incentive scheme that applies to each study arm.

**Table 1:** Financial incentives per study arm

Study arm	Contingent incentives	Non-contingent payment	Assessment incentives	Total payments
Control	None	Yes (SGD75 paid at month 6)	SGD15 at each study assessment (baseline, month 3 and month 6)	Non-contingent payment: SGD75 Assessment payments: SGD45 Total: SGD120
Process	SGD3.50 weekly: for measuring blood glucose on 3 non-consecutive days/week SGD0.50 daily: for taking medication daily as prescribed SGD1 daily: for regular physical activity (8000 steps/day)	None	SGD15 at each study assessment (baseline, month 3 and month 6)	Maximum incentive payments: SGD336 Assessment payments: SGD45 Total: SGD45 to SGD381
Outcome	SGD2 weekly for 1 glucose reading per week being in normal range (between 4 to 7 mmol/L) SGD7 weekly for 2 glucose readings per week being in normal range (between 4 to 7 mmol/L) SGD14 weekly for 3 glucose readings per week being in normal range (between 4 to 7 mmol/L)	None	SGD15 at each study assessment (baseline, month 3 and month 6)	Maximum incentive payments: SGD336 Assessment payments: SGD45 Total: SGD45 to SGD381

SGD 1 = 0.56 GBP and USD 0.72 on 6 June 2017

#### Arm 1: Usual care (UC)

SingHealth Polyclinics have a structured framework to care for people with diabetes. Team-based care which involves the physicians, nurses, laboratory staff and pharmacists, is delivered. The model of care includes consults by the nurse clinician service and telecare service for patients with good glycaemic control; and family physician clinics, multidisciplinary teams and case managers for people with suboptimally controlled diabetes.

All new diabetes patients are referred to an in-house health counsellor for counselling and advice. The education on diabetes is done in a graded manner over four to six visits. All diabetes patients are then followed up by the physicians and nurses at 2- to 4-monthly intervals, depending on their glycaemic control. Further education by nurses is done as needed for these visits. There is an in-house laboratory at all SingHealth Polyclinics, which makes it possible to test HbA1c levels on arrival before the patient’s appointment with the physician. In addition to the follow-up consultations with polyclinic physicians, the patients are sent for annual eye and foot screening done by trained nurses. There are also in-house pharmacists who assist patients in understanding their medication doses and regimens.

In order to properly identify the effect of contingent financial incentives, we introduced slight deviations to UC. First, the participants in the UC arm will receive a Participant Leaflet (Additional files 2 and 3) reminding them of the following recommended guidelines:

- Participants should aim to have blood glucose levels between 4 and 7 mmol/L before meals
- The best time for participants to test their blood glucose is before breakfast
- Participants should measure their blood glucose levels on at least three non-consecutive days per week
- Participants should aim to take 8000 steps every day
- Participants should take their diabetes medication(s) as recommended each day

Participants in the UC arm will also benefit from the assessment of their treatment adherence during the month-3 and monh-6 visits even though they cannot earn incentives for being adherent to the recommendations.

#### Arm 2: Process incentive

Participants in the process incentive arm will receive all the elements of UC described above and, in addition, will have the opportunity to earn financial incentives contingent on meeting specified process goals as stated below. These are also stated in the Participant Leaflet given to participants in the process incentive arm (Additional files 4 and 5):

- *SGD3.50 weekly for blood glucose testing*: measuring blood glucose on three non-consecutive days each week. This will be assessed via timestamps logged by the glucometer. For the Process Incentive arm, testing counts towards the goal even when readings fall outside the recommended range. The incentive amount approximately offsets the cost of the required glucometer strips and lancets
- *SGD0.50 daily for medication adherence*: taking all medications as prescribed during the day, which will be monitored by the medication tracker. This will be assessed based on medication-taking times within specified time windows. For instance, if a participant’s specified timing is 5 a.m. to 11 a.m. (breakfast) and 5 p.m. to 11 p.m. (dinner), a reading has to be logged within both windows for the participant to be considered adherent on that day. For the sake of simplicity, the incentive amount was set at the same level as for glucose testing
- *SGD1.00 daily for regular physical activity*: taking 8000 steps during the day as recorded by the pedometer. The incentive amount is the double of that for glucose testing and medication adherence to account for the relative difficulty of the goal to achieve

Overall, participants can receive incentives worth up to SGD14 a week amounting to SGD336 over two 3-month (12 weeks) periods.

#### Arm 3: Outcome incentive

Participants in the outcome incentive arm will receive all the elements of UC described above and, in addition, will have the opportunity to earn financial incentives contingent on meeting health outcomes goals. Specifically, these participants will earn financial incentives for recording glucose readings within the normal range (between 4 to 7 mmol/L) before a meal on three non-consecutive days within the week as stated below. These are also stated in the Participant Leaflet given to participants in the outcome incentive arm (Additional files 6 and 7).

- SGD2 weekly if one glucose reading falls within the normal range
- SGD7 weekly if two glucose readings fall within the normal range
- SGD14 weekly if three glucose readings fall within the normal range

If a participant tests more than once during a day, only the first measurement will count. If a participant tests on a consecutive day, this day will not count towards the goal. Overall participants can receive incentives worth up to SGD14 a week amounting to SGD336 over two 3-month (12 weeks) periods. Note that the incentive amount was set at the same level as for the process incentive arm so that to control for incentive size.

### Outcome measures

#### Primary outcome

The primary outcome is *mean change from baseline in glycated haemoglobin HbA1c at month 6*. HbA1c is an intermediary diabetes outcome that is commonly used in the assessment of diabetes management interventions [11] and that has been shown to be strongly associated with diabetes-related complications and mortality [20].

#### Secondary outcomes

- *Mean number of blood glucose days at month 6*. Up to three non-consecutive testing days during the last week of the intervention will be counted. This outcome corresponds to UC’s recommendations and to one of the goals that is incentivised in the process incentive arm
- *Mean number of glucose readings that fall between 4 and 7 mmol/L at month 6*. This outcome is consistent with UC’s recommendations for blood glucose testing before meals. Consistent with the outcome incentive arm’s goal, only the first reading recorded on a maximum of three non-consecutive days will be taken into consideration during the last week of the intervention
- *Mean number of medication-adherent days at month 6*. Medication-adherent days are defined as days where all medication doses were taken during pre-defined time windows as verified by the medication tracker. The number of medication-adherent days during the last week of the intervention will be calculated for each participant. This outcome corresponds to UC’s recommendation and to one of the goals that is incentivised in the process incentive arm
- *Mean number of physically active days at month 6*. The number of days where the pedometer records at least 8000 steps during the last week of the intervention will be calculated for each participant. This outcome corresponds to UC’s recommendations and to one of the goals that is incentivised in the incentives arm.
- *Mean cost of financial incentives at month 6.* This outcome will be calculated as the total contingent financial incentives earned by process incentive and outcome incentive arm participants during the last week of the intervention. This outcome will be used as part of the cost-effectiveness analysis
- *Mean time taken by the intervention at month 6.* This outcome will be calculated as the total number of minutes spent by the CRC on adherence calculation and payment of financial incentives for process incentive and outcome incentive arms participants during the month-6 assessment. This outcome will be used as part of the cost-effectiveness analysis

#### Explanatory outcomes

- *Mean change from baseline in glycated haemoglobin HbA1c at months 12 and 18*
- *Proportion of participants whose oral medication was titrated up at months 6, 12 and 18*
- *Proportion of participants who switched to insulin therapy at months 6, 12 and 18*
- *Mean change from baseline in EQ-5D-5L* [21] *score at month 6* as a measure of functional health status. This scale has been validated in people with type-2 diabetes [22]. In Singapore, both the English and Mandarin versions have been validated in people with cancer [23]
- *Mean change from baseline in Brief Illness Perception Questionnaire* [24] *(BIPQ) score at month 6*. This scale has been validated in English for multiple health conditions including diabetes [25]. The Mandarin version has been validated in Taiwan in people with coronary heart disease [26]
- *Mean change from baseline in Self-Monitoring of Blood Glucose* [27] *(SMBG) score at month 6*. This scale has been validated in English in people with type-2 diabetes but has yet to be validated in Mandarin and in Singapore
- *Mean change from baseline in general and specific scores of the* Beliefs about Medication Questionnaire [28] *(BMQ) at month 6*. The English version of the scale has been validated for several chronic diseases including diabetes [29] while the Mandarin version has been validated in other settings such as after mechanical heart-valve replacement [30] and depression [31]. Validity in Singapore has not been established for this scale
- *Mean change from baseline in the exercise subscale of the Diabetes Self-Care Activities* [32] *(DSCA) at month 6.* The English version of the scale has been pretested for acceptability and comprehensibility in a study involving people with type-2 diabetes [32] but has neither been pretested in Mandarin nor in Singapore

### Sample size

A key parameter is the variability in post-intervention HbA1c levels. To assess this, we have used data from a pilot intervention aimed at improving diabetes outcomes through a comprehensive educational and management programme (that did not include financial incentives). Among 54 participants with suboptimally controlled diabetes, we found that the standard deviation in participant HbA1c levels was 1.29 at baseline and 1.99 after a 3-month intervention. Based on this data, we decided to use a standard deviation of 2 for the variability in post-intervention HbA1c levels to calculate the sample size for this trial.

We have first computed the size of the intervention arms that is required for testing whether there is a statistical difference between post-intervention HbA1c levels between the outcome and process incentive arms (primary objective 2). After applying a Bonferroni correction by dividing the test’s significance level by 2 (which is the number of primary hypotheses to test in this trial), we found that 76 participants per intervention arm are necessary to detect mean differences in HbA1c levels of 1% between these study arms (with significance level of 5/2 = 2.5% and 80% power). Given the resulting cumulated sample size for the intervention arms of 76 + 76 = 152, we then computed the size required for the UC arm to test the overall effect of financial incentives (primary objective 1) with the same effect size, significance level, and power. This computation yielded a sample size of 51 for the UC arm. After accounting for approximately 20% attrition in each study arm, we set the final sample size at 60 for the UC arm and 90 for each intervention arm, for a grand total of 240 participants.

### Randomisation

Prior to the start of recruitment, randomisation numbers will be generated by the principal investigator of the study and the project coordinator using Stata 13.2 to create an assignment schedule for block-randomisation to allocate eligible participants into one of the three study arms in a ratio of 2:3:3. Whether participants are current glucometer users or not (current vs. new glucometer users) will be used as the blocking factor, and the block size will not be communicated to study site in order to minimise the predictability of the random sequence. The project coordinator and the principal investigator of the study will then store the assignment schedule (including generation information) on a secure server at Duke-NUS. For allocation concealment, the project coordinator and a witness external to the study will enclose the assignments in sequentially numbered, opaque, sealed, randomisation envelopes and the project coordinator will hand these to the CRC along with other relevant study materials. Note that the behavioural nature of the intervention precludes masking the arm allocation to both the study team and participants. The allocation will be revealed to both the CRC and participant upon enrolment. However, the study arm assignment will be not be revealed to the laboratory staff assessing the primary outcome (HbA1c test).

### Participant recruitment, retention, withdrawal and discontinuation

Participants will either be referred by a physician from the Family Physician Clinic and General Clinic at Geylang Polyclinic, or recruited through posters advertising the study at the nine SingHealth Polyclinics in Singapore and through newspaper advertising. Prospective participants will call the CRC to schedule an appointment. If a patient is referred by a physician, the physician will verify that the patient fulfils the basic study eligibility criteria prior to referring the patient to the CRC. The CRC will either start screening the prospective participant immediately or schedule a research appointment within 2 weeks of the physician referral or at the patient’s next doctor appointment at the clinic (if any) depending on the patient’s availability. When the CRC administers the screener questionnaire, if a prospective participant answers ‘yes’ to any of the PARQ (Physical Activity Readiness Questionnaire) questions the patient will require approval from a physician before joining the study. If the patient is eligible the CRC will conduct the informed consent process and enrol the patient on to the study.

All participants will receive text messages encouraging them to remain in the study. These messages will be sent 7 days after the baseline visit as well as 28 and 56 days after the baseline and month-3 visits. Table 2 describes the text messaging schedule for all three study arms. Table 3 lists the corresponding text messages which will be automatically sent by the web application after the CRC has registered the participant during the baseline visit. To avoid any bias, all participants will receive the same number of text messages and the messages will be strictly identical except for the description of the financial incentive which is arm-specific. In addition, lost and broken devices will be replaced free of charge to enable continued participation in the study.

**Table 2:** Text messaging schedule

Time point	SMS reminder ID		
	Arm 1	Arm 2	Arm 3
Week 1	1	1	1
Month 1	2–1	2–2	2–3
Month 2	3–1	3–2	3–3
Month 4	2–1	2–2	2–3
Month 5	3–1	3–2	3–3
Day 21 of each month where no step activity is recorded	4	4	4

**Table 3:** List of text messages to be sent to the participants

ID	Text message
1	Dear participant, many thanks for taking part in the Trial to Incentivise Adherence for Diabetes (TRIAD) study. Please remember that regular physical activity, taking medication as recommended and monitoring your glucose level will help manage your diabetes. For assistance, please contact (clinical research coordinator (CRC’s) name) at (CRC’s contact)
2–1	Dear TRIAD participant, please remember that regular physical activity, taking medication as advised and glucose level monitoring will help manage your diabetes. You will receive SGD15 for attending the month-3 visit. You will also receive SGD90 at the end of the study for recording your data and attending the month-6 visit. For assistance, please contact (CRC’s name) at (CRC’s contact)
2–2	Dear TRIAD participant, please remember that regular physical activity, taking medication as advised and glucose level monitoring will help manage your diabetes. You will earn SGD14 weekly for meeting all activity, medicine-taking and glucose testing goals. For assistance, please contact (CRC’s name) at (CRC’s contact)
2–3	Dear TRIAD participant, please remember that regular physical activity, taking medication as advised and glucose level monitoring will help manage your diabetes. You will earn SGD14 weekly for meeting your glucose level goals. For assistance, please contact (CRC’s name) at (CRC’s contact)
3–1	Dear TRIAD participant, please remember that regular physical activity, taking medication as advised and glucose level monitoring will help manage your diabetes. You will receive SGD90 at the end of the study for recording your data and attending the month-6 visit. For assistance, please contact (CRC’s name) at (CRC’s contact)
3–2	Dear TRIAD participant, please remember that regular physical activity, taking medication as advised and glucose level monitoring will help manage your diabetes. You will earn SGD14 weekly for meeting all goals. For assistance, please contact (CRC’s name) at (CRC’s contact)
3–3	Dear TRIAD participant, please remember that regular physical activity, taking medication as advised and glucose level monitoring will help manage your diabetes. You will earn SGD14 weekly for meeting your goals. For assistance, please contact (CRC’s name) at (CRC’s contact)
4	Dear TRIAD participant, a gentle reminder that if you have not yet synced your Fitbit^TM^ you have 1 week left to sync. If you wish you can sync at the polyclinic, please contact (CRC’s name) at (CRC’s contact). Thank you

Participants will be free to withdraw their consent and discontinue their participation at any time. When possible, the CRC will administer a short exit questionnaire to inquire about the reasons of the withdrawal and collect the study devices from the participants. Further, participants will be discontinued from the study if the site principal investigator decides that continuing their participation could be harmful, or if the participant becomes pregnant. Additionally, if during the study, the participant’s condition is deemed unsatisfactory the physician may advise the patient to begin using insulin. In this case the patient will no longer be eligible for the study and will be discontinued. Participants who are discontinued from the study will receive SGD45 in compensation for forgoing potential payments that the patient might have received had they remained in the study.

### Data collection

To measure HbA1c levels for all participants, blood tests will be carried out by laboratory staff at the polyclinic at baseline and month 6. The procedure for blood tests will follow the usual polyclinic process. One of the physicians at the polyclinic will order the blood test and give a printed form to the patient. The patient will arrange and attend an appointment at the polyclinic laboratory. The laboratory technician will obtain a capillary specimen from the patient and will perform the HbA1c test on-site via the Siemens DCA Vantage® Analyzer using immunoassay. The costs of these tests will be covered by the study.

The data from the glucometers will be collected at months 3 and 6 during the clinic visits. As glucose testing is part of UC, all patients receive support for adequate usage of their glucometer. For most glucometers in use at the polyclinic (this includes the Accu-Chek Performa™, the Accu-Chek Active™ and the Nipro TRUEresult™) the device will be scanned using the appropriate docking station and the data will be downloaded to the relevant glucometer software. From the software, the data will be exported to an Excel file where the CRC will check the validity of the data. The Excel file will then be imported to the trial application where treatment adherence and the corresponding incentives will be calculated. For the less common glucometers, the data stored on the device will be manually entered by the CRC onto an Excel file which will then be imported into the trial application.

The data from the medication trackers will also be collected at months 3 and 6. The CRC will scan each medication tracker using the CertiScan™ desktop reader and the data will be downloaded to the Med-ic™ software. From the software the data will be exported to an excel file where the CRC will check the validity of the data. The Excel file will then be uploaded to the trial application where medication adherence and the corresponding financial incentives will be calculated.

Each pedometer will be linked to an anonymous online Fitbit™ account that will be created for the study. The trial application will automatically synchronise with the Fitbit™ accounts and automatically update physical activity data for adherence and incentives calculations. This data will be checked regularly by the project coordinator to verify that the participants are synchronising their pedometer with their Fitbit™ account and that this data gets transmitted to the trial application. This is important because the pedometers can store up to 30 days of data. Participants who do not synchronise for 21 days will receive an automatic text message to remind them to synchronise. Participants who do not have access to a smartphone or personal computer will need to come to the polyclinic once a month where the CRC will synchronise their pedometers for them.

Paper-based survey questionnaires will be administered at baseline and month 6 by the CRC. Both questionnaires include the European Quality of Life-5 Dimensions-5 Levels,(EQ-5D-5L), BIPQ and BMQ survey instruments. The baseline survey also includes patient socioeconomic characteristics while the month-6 questionnaire contains questions on compliance with the medication tracker and medication-purchasing habits during the intervention period.

In addition, the CRC will complete participant checklists at baseline, months 3, 6 and 18. The Baseline checklist will include the participant’s current HbA1c reading and the date of the blood test. This information will be obtained from the SingHealth computerised patient database at Geylang Polyclinic. The randomisation code and study arm, the participants’ diabetes medication regimen, and whether the participants are new or current glucometer users will also be recorded on the checklist. The CRC will also confirm the participants have understood which study arm they have been randomised to, record the response on the checklist and explain again if necessary.

At months 3 and 6, the CRC will again check that the participant understands the goals and rewards of the study arm they have been randomised to, record the response on the corresponding checklist and re-explain if necessary. Details on fasting which could affect medication dosages and times; overseas trips which involve time zone changes that will possibly impact the study devices; and hospitalisations will also be recorded at months 3 and 6. Further, the participants’ adherence and payments made will be recorded along with the time spent by the CRC on adherence calculation and corresponding incentive payments. Last, the CRC will record any changes to the participants’ diabetes medication regimen based on their medical records.

At month 18, the CRC will complete a checklist solely from the patient’s medical records. This checklist will include recording HbA1c results and any changes in medication regimen since the patient completed the intervention.

### Data management and monitoring

The CRC will check the completeness of the study documents at the polyclinic before transferring them to Duke-NUS. Such transfers will be acknowledged and documented in a log file maintained by the study team. To ensure confidentiality, only de-identified research data will be passed to Duke-NUS. Study team members at Duke-NUS will digitise the responses from the paper-based questionnaires and checklists into a data entry Excel file. Data will be double-entered for accuracy by two separate members of the Duke-NUS team. To further minimise errors, the data entry sheets have been designed to only accept values that are within the correct range.

Once the first 80 participants have successfully completed the month-6 assessments, the following outcomes will be assessed by a trained statistician: HbA1c levels and adherence across physical activity, medication and blood glucose testing rates and levels. These will be repeated when 160 participants complete the month-6 assessment. Further, attrition and missing data patterns will be analysed. The purpose of these interim analyses is to detect potential issues that might have arisen during the data collection process before the end of the trial and to report preliminary results to SingHealth Centralised Institutional Review Board (CIRB) on an annual basis. No stopping rules have been defined for this trial.

The CRC will ask participants about potential adverse events. If a patient has been hospitalised during the intervention, the CRC will record the details. Reporting of adverse events involves notifying the SingHealth CIRB and submitting the Serious Adverse Events (SAE) Reporting Form within the stipulated timeframe. The notifying and reporting requirements depend on the severity, nature and causality of the event and there are specific procedures that must be followed [33].

No data monitoring committee will be used for this trial. UC is consistent with the existing standard of care at the SingHealth Polyclinics. Additional elements, such as self-paced physical activity and financial incentives, do not involve more than minimal risk to the participants. This trial is subject to study review visits and/or audits by the SingHealth Research Quality Assurance unit which is responsible for ensuring that all investigator-initiated research processes are conducted suitably, adequately, effectively and efficiently across the SingHealth cluster. These study review visits/audits may be conducted routinely, triggered by CIRB or upon an investigator’s request.

### Statistical methods

#### Primary analysis

Change from baseline in HbA1c levels at month-6 will be linearly regressed on (1) binary variables indicating the intervention arms with UC as a reference category, (2) baseline HbA1c levels, (3) a binary variable indicating titration change and (4) other baseline characteristics (gender, age, language spoken, education, occupation, income, EQ-5D-5L, BIPQ, SMBG, BMQ and DSCA scores, and binary variables indicating comorbidities) in order to increase statistical efficiency. The analysis will be performed according to an intention-to-treat approach. All missing data will be imputed using Markov chain Monte Carlo multiple imputation. To test the robustness of the results, we will carry forward the last measured HbA1c level (baseline or month 3) for those participants who withdraw from the study as those intervention participants who do not achieve their goals might disproportionally withdraw from the study. The last measured HbA1c level will also be carried forward for those participants who are discontinued from the study after switching from oral to insulin therapy.

#### Secondary analyses

The number of blood glucose testing days, glucose readings within acceptable range, medication-adherent days, and physically active days at month 6 will be linearly regressed on binary variables indicating the intervention arms and on the same baseline characteristics as for the primary analysis. If the interventions show statistically significant improvements in HbA1c levels, we will conduct a cost-effectiveness analysis for both intervention arms. The perspective of the cost-effectiveness analysis will be that of the health system. Effectiveness will be measured by the primary outcome and costs will include the average financial incentives paid, labour costs associated to the administration of the incentives, and the cost of devices. The incremental cost-effectiveness ratio between each intervention and UC will be calculated and compared to those of other interventions aiming at reducing HbA1c levels.

### Explanatory analyses

The same model as for the primary analysis will be conducted with changes from baseline in HbA1c levels at months 12 and 18. Logistic regressions of binary variables indicating whether the participants have had a titration change of their oral medicine, and whether they have switched to insulin therapy at months 6, 12 and 18 will be estimated. Linear regressions of the changes from baseline in EQ-5D-5L, BIPQ, SMBG, BMQ and DSCA scores at month 6 will also be estimated. For all models, the covariates will be binary variables indicating the intervention arms and the same other baseline characteristics as for the primary analysis. Next, the models used in the primary and secondary analyses will be extended by adding interaction terms between potential intervention moderators (gender, age, language spoken, education, occupation, income, EQ-5D-5L, BIPQ, SMBG, BMQ and DSCA scores, and binary variables indicating comorbidities) and the binary variables indicating study arms. In particular, this analysis will be used to determine the benefit incidence of the intervention according to socioeconomic factors.

### Ethics and dissemination

This study has been approved by the SingHealth Centralised Institutional Review Board E (Ref 2013/830/E) which oversees family medicine research at SingHealth. The principal investigator is responsible for informing the CIRB of any amendments to the protocol or other study-related documents, as per local requirements.

The consent process will be carried out at the SingHealth Polyclinic in Geylang. Interested and potentially eligible patients will be referred for the study by a physician at the polyclinic. Alternatively, patients may respond to study posters or newspaper advertisements and contact the CRC directly. In all cases the CRC will explain the study to the patient in either English or Mandarin. The Participant Information Sheet and Consent Form (see Additional files 8 and 9), and all other participant documents will be available in both English and Mandarin. Patients are expected to be able to give consent to participate in the study on their own. No arrangements have been made for informed consent to be taken from a legally acceptable representative of the patient. No provisions have been made to compensate participants for research-related injuries as the study does not involve more than minimal risks. However, compensation may be considered on a case-by-case basis for unexpected injuries due to non-negligent causes.

A unique participant ID will be assigned to all patients who are successfully enrolled on the study. Study questionnaires and checklists will only refer to the participants using this ID number. In addition, data is de-identified before is it passed to Duke-NUS. Records containing identifiable data, such as the screener and Consent Form, will be stored in locked cabinets at the polyclinic with restricted access. All study materials, such as study devices, will be kept in locked cabinets at the polyclinic and Duke-NUS. Only the investigators and authorised personnel directly involved with the study will have access to the data. All data files will be password protected and stored on a secure server at Duke-NUS for 10 years and then securely destroyed.

Investigators will have unrestricted access to the research data upon completion of the trial. Main trial results will be published irrespective of the magnitude and direction of the effects and their statistical significance. To be listed as authors, investigators will have to meet all four conditions for authorship recommended by the International Committee of Medical Journal Editors (ICMJE) [33]. Those investigators and collaborators who do not meet all four criteria will be acknowledged for their contribution.

## Discussion

The primary objectives of this randomised controlled trial are to determine whether adding financial incentives to UC can improve HbA1c levels among people with suboptimally controlled diabetes in a primary-care setting; and to determine whether financial incentives should be directed at processes or at achieving intermediary health outcomes. In order to meet these objectives it will be crucial that participants fully understand the goals and rewards of their study arm. First, participants must not confuse the non-contingent incentives received to compensate for their time taking part in the trial with the process and outcome incentives that participants in the intervention arms can receive if they meet their goals. Second, it is important to make sure that participants understand the calculation of the incentive amount that they can expect to receive based on their diabetes self-management efforts. Great care will be taken by the CRC to explain the incentive scheme during the baseline visit. In addition, the CRC will check whether the participants remember the incentive calculation at the month-3 and month-6 visits, will re-explain the calculations if necessary, and will record whether the calculations were understood in order to assess the quality of the study. The Participant Leaflets also contain a detailed section on incentive goals and calculation for the study arm the participants have been randomly assigned to.

The possibility of gaming has also been considered. In order to earn incentives without improving their diabetes management, some participants might, for instance, ask a third person to use their device, shake their pedometer to gain extra steps, and open and close their tracking device without taking their medication. In efforts to contain gaming, participants will be asked to sign a participation oath which has been shown to decrease the likelihood of cheating in other studies [34] and has been applied to financial incentives studies [15, 35]. Furthermore, several quality checks will be performed. Participants will be asked to keep all their medication bills and bring them to the clinic visits. In order to assess the validity of pedometer data, the distribution of steps within days will be examined to detect implausible patterns. Glucometer readings data will be compared to HbA1c levels in efforts to detect implausible discrepancies. It is worth noting that, if present, gaming would not affect the primary analysis as it is based on HbA1c tests.

We will first compare our primary results to the only other incentive study that reports HbA1c levels as a primary outcome [18]. We will then compare our results to those found in other types of diabetes management interventions such as education [7], blood glucose self-monitoring alone [8], free-living physical activity [9], computer-based interventions [10] and quality improvement strategies [11]. This study will provide evidence on whether financial incentives can cost-effectively improve diabetes management. It will also be important to determine who benefits the most from the intervention. By interacting socioeconomic characteristics with the intervention effect, this study will provide evidence on the benefit incidence of interventions involving financial incentives. Last, by comparing process to outcome incentives, this study will inform the design of future incentive strategies in chronic disease management and beyond.

## Trial status

Recruitment to the TRIAD study began in February 2015 and is ongoing. As of 31 May 2017, 140 participants (58.3% of the total sample size) have been enrolled in the study.

## Additional files

Additional file 1: SPIRIT Checklist. (DOC 126 kb)

Additional file 2: Excerpt of TRIAD Participant Instruction Booklet (UC – English). This booklet is given to all English-speaking UC participants when they join the study. The booklet reminds the participants of the diabetes management recommendations and provides information on the use of the study devices. (PDF 218 kb)

Additional file 3: Excerpt of TRIAD Participant Instruction Booklet (UC – Mandarin). This booklet is given to all Mandarin-speaking UC participants when they join the study. The booklet reminds the participants of the diabetes management recommendations and provides information on the use of the study devices. (PDF 410 kb)

Additional file 4: Excerpt of TRIAD Participant Instruction Booklet (Process Incentive – English). This booklet is given to all English-speaking Process Incentive arm participants when they join the study. The booklet reminds the participants of the diabetes management recommendations, gives detailed explanations of the goals and incentives for this study arm, and provides information on the use of the study devices. (PDF 228 kb)

Additional file 5: Excerpt of TRIAD Participant Instruction Booklet (Process Incentive – Mandarin). This booklet is given to all Mandarin-speaking Process Incentive arm participants when they join the study. The booklet reminds the participants of the diabetes management recommendations, gives detailed explanations of the goals and incentives for this study arm, and provides information on the use of the study devices. (PDF 352 kb)

Additional file 6: Excerpt of TRIAD Participant Instruction Booklet (Outcome Incentive – English). This booklet is given to all English-speaking Outcome Incentive arm participants when they join the study. The booklet reminds the participants of the diabetes management recommendations, gives detailed explanations of the goals and incentives for this study arm, and provides information on the use of the study devices. (PDF 227 kb)

Additional file 7: Excerpt of TRIAD Participant Instruction Booklet (Outcome Incentive – Mandarin). This booklet is given to all Mandarin-speaking Outcome Incentive arm participants when they join the study. The booklet reminds the participants of the diabetes management recommendations, gives detailed explanations of the goals and incentives for this study arm, and provides information on the use of the study devices. (PDF 351 kb)

Additional file 8: Participant Information Sheet and Consent Form (English). All English-speaking participants will complete this Consent Form before joining the study. The participant signs two copies, one copy is kept by SingHealth Polyclinics and one copy is given to the participant. (PDF 438 kb)

Additional file 9: Participant Information Sheet and Consent Form (Mandarin). All Mandarin-speaking participants will complete this Consent Form before joining the study. The participant signs two copies, one copy is kept by SingHealth Polyclinics and one copy is given to the participant. (PDF 562 kb)

## Abbreviations

BIPQ: Brief Illness Perception Questionnaire
BMQ: Beliefs about Medication Questionnaire
CIRB: Centralised Institutional Review Board
DSCA: Diabetes Self-Care Activities
EQ-5D-5L: European Quality of Life-5 Dimensions-5 Levels
HbA1c: Glycated haemoglobin
PARQ: Physical Activity Readiness Questionnaire
SAE: Serious Adverse Events
SGD: Singapore dollar
SMBG: Self-Monitoring of Blood Glucose
TRIAD: Trial to Incentivise Adherence for Diabetes
UC: Usual care
