# Supplementary material for: Trial to Incentivise Adherence for Diabetes (TRIAD): study protocol for a randomised controlled trial
Source: Trials. 2017 Nov 17;18:551. doi: 10.1186/s13063-017-2288-6 (PMC5693491; doi:10.1186/s13063-017-2288-6)
Supplement: Supplementary file 2 — Excerpt of TRIAD Participant Instruction Booklet (UC – English). This booklet is given to all English-speaking UC participants when they join the study. The booklet reminds the participants of the diabetes management recommendations and provides information on the use of the study devices. (PDF 218 kb) [file 13063_2017_2288_MOESM2_ESM.pdf]

Recommendations to optimally manage your diabetes

- Aim to have blood sugar levels **between 4.0 and 7.0mmol/L** before your meals.
- Measure your blood sugar levels at least on **3 non-consecutive days** in the week.
- The best time to test your blood sugar is before you take your breakfast.
- You are also recommended to test your blood sugar 2 hours after your meals.
    - readings should be below 8.5 mmol/L if you have type 2 diabetes.
- Aim to take **8,000 steps** every day.
- Take your diabetes medication(s) as recommended each day.
- The Site Study Coordinator will note in the table below when you should take your medication:

Name of diabetes medication	Breakfast	Lunch	Dinner

Fairness Payment

- You will receive a fairness payment of $75 at the end of the study if you fulfill all study requirements, irrespective of following the recommendations.

Additional information

- Your first monitoring assessment period will start the day after your Baseline visit. This monitoring period will be for 84 days.
- Your second monitoring assessment period will start the day after your month 3 visit. This monitoring period will also last 84 days.
- Please bring the 3 study devices (Fitbit, eCAP and glucometer) to your Month 3 and Month 6 assessments.
- You will receive $15 when you join the study, $15 for completing the Month 3 Visit, and $15 for completing the Month 6 Visit.
- Payments will be made in the form of NTUC vouchers.
- You will be required to return the Fitbit and eCAP at the end of the study. Participants who do not have their own glucometer and are given a glucometer at the beginning of the study may keep the glucometer after the study has finished if they wish.
