# Supplementary material for: Trial to Incentivise Adherence for Diabetes (TRIAD): study protocol for a randomised controlled trial
Source: Trials. 2017 Nov 17;18:551. doi: 10.1186/s13063-017-2288-6 (PMC5693491; doi:10.1186/s13063-017-2288-6)
Supplement: Supplementary file 3 — Excerpt of TRIAD Participant Instruction Booklet (UC – Mandarin). This booklet is given to all Mandarin-speaking UC participants when they join the study. The booklet reminds the participants of the diabetes management recommendations and provides information on the use of the study devices. (PDF 410 kb) [file 13063_2017_2288_MOESM3_ESM.pdf]

最适合治疗您的糖尿病的建议

- 目标是在吃饭前，血糖值应该要在4.0 至 7.0 mmol/L之间。
- 一个星期里在**三个非连续天**测量您的血糖值。
- 最好测量血糖值的时间是在吃早餐前。
- 我们也建议您吃饭后两个小时，测量您的血糖值。
  ◊您的血糖应该在8.5mmol/L 以下要是您有2型糖尿病

- 目标是每天走8,000步。
- 服用每天所推荐的糖尿病药物。

- 研究分支联络人会在研究以下的表写您应该几时吃您的药物:

糖尿病药物的名称	早餐	午餐	晚餐

公平 付款

- 您的第一次监测评估期将从您的基线访问后的一天开始。这一监测期将是84天。

- 您的第二次监测评估期将从您第3个月访问后的一天开始。该监控期也将持续84天。

- 要是您有达到全部研究的要求，不管您有没有照以上的建议，这个研究完成过后，您会收到$75的公平补贴。

- 请带来3研究设备（Fitbit和eCAP和血糖测计仪）到你的第3个月和第6个月评定。

- 您会因参加这项研究，完成第三个月和第六个月的评估而各获得$15的礼券。

- 付款是NTUC的礼券。

- 研究完成过后，您必须退回您拿到的Fitbit和eCAP.

- 如果参加者没有自己的血糖测计仪跟他在研究开始时拿到一个血糖测计仪，如果他想要研究过后可以留给自己使用。
