# Supplementary material for: Trial to Incentivise Adherence for Diabetes (TRIAD): study protocol for a randomised controlled trial
Source: Trials. 2017 Nov 17;18:551. doi: 10.1186/s13063-017-2288-6 (PMC5693491; doi:10.1186/s13063-017-2288-6)
Supplement: Supplementary file 4 — Excerpt of TRIAD Participant Instruction Booklet (Process Incentive – English). This booklet is given to all English-speaking Process Incentive arm participants when they join the study. The booklet reminds the participants of the diabetes management recommendations, gives detailed explanations of the goals and incentives for this study arm, and provides information on the use of the study devices. (PDF 228 kb) [file 13063_2017_2288_MOESM4_ESM.pdf]

Recommendations to optimally manage your diabetes

- Aim to have blood sugar levels **between 4.0 and 7.0mmol/L** before your meals.
- Measure your blood sugar levels at least on **3 non-consecutive days** in the week.
- The best time to test your blood sugar is before you take your breakfast.
- You are also recommended to test your blood sugar 2 hours after your meals.
    - readings should be below 8.5 mmol/L if you have type 2 diabetes.
- Aim to take **8,000 steps** every day.
- Take your diabetes medication(s) as recommended each day.
- The Site Study Coordinator will note in the table below when you should take your medication:

Name of diabetes medication	Breakfast	Lunch	Dinner

Goals and financial incentives

Take your meds, walk your steps, test your blood

You can earn financial incentives if you meet the following goals:

- SGD **3.50** weekly for testing your blood sugar levels on **3 non-consecutive days** in the week as recorded by your glucometer.
    - Testing counts towards your goal even when readings fall outside the recommended range.
    - In case you test more than once during a day, this will only count as one measurement.
    - In case you test on a consecutive day, this day will not count towards your goal. For instance, if you test on Wednesday, testing on Thursday will not count towards your goal.

- SGD **0.50** daily for taking your medication as recommended and as recorded by your eCAP.
    - In order to be counted towards your goal, you will need to take your diabetes medication at the appropriate mealtime.

- SGD **1.00** daily for taking **8,000 steps** as recorded by your Fitbit Zip.

If you meet your goals you can receive incentives worth up to SGD **14** per week.

Calculations and payment of financial incentives

- The Site Study Coordinator will calculate the incentives by checking your data on the study devices (i.e. Fitbit, eCAP and glucometer).
- Please bring the 3 study devices (Fitbit, eCAP and glucometer) to your Month 3 and Month 6 assessments.
- All payments will be made in the form of NTUC vouchers at your month 3 and 6 assessments (or at the next visit if the incentive cannot be computed during the visit).
- <u>Payment will only be made if data recorded by study devices is provided</u>.

Additional information

- Your first monitoring assessment period will start the day after your Baseline visit. This monitoring period will be for 84 days. Your incentives will be calculated based on this 84 day period.

- Your second monitoring assessment period will start the day after your month 3 visit. This monitoring period will also last 84 days. Your incentives will be calculated based on this 84 day period.

- You will receive $15 when you join the study, $15 for completing the Month 3 Visit, and $15 for completing the Month 6 Visit.

- You will be required to return the Fitbit and eCAP at the end of the study.

- Participants who do not have their own glucometer and are given a glucometer at the beginning of the study may keep the glucometer after the study has finished if they wish.
