# Supplementary material for: Trial to Incentivise Adherence for Diabetes (TRIAD): study protocol for a randomised controlled trial
Source: Trials. 2017 Nov 17;18:551. doi: 10.1186/s13063-017-2288-6 (PMC5693491; doi:10.1186/s13063-017-2288-6)
Supplement: Supplementary file 5 — Excerpt of TRIAD Participant Instruction Booklet (Process Incentive – Mandarin). This booklet is given to all Mandarin-speaking Process Incentive arm participants when they join the study. The booklet reminds the participants of the diabetes management recommendations, gives detailed explanations of the goals and incentives for this study arm, and provides information on the use of the study devices. (PDF 352 kb) [file 13063_2017_2288_MOESM5_ESM.pdf]

最适合治疗您的糖尿病的建议

- 目标是在吃饭前，血糖值应该要在4.0 至 7.0 mmol/L之间。

- 一个星期里在**三个非连续天**测量您的血糖值。

- 最好测量血糖值的时间是在吃早餐前。

- 我们也建议您吃饭后两个小时，测量您的血糖值。

◊您的血糖应该在8.5mmol/L 以下要是您有2型糖尿病

- 目标是每天走8,000步。
- 服用每天所推荐的糖尿病药物。
- 研究分支联络人会在研究以下的表写您应该几时吃您的药物:

糖尿病药物的名称	早餐	午餐	晚餐

目标和金钱奖励

吃药，步行，测试你的血液

您将获得奖励要是您达到以下的目标：

- 每周3.50新元，要是您在一周之内3个非连续天使用您的血糖仪测量您的血糖值。
  - ◊ 试验计数朝着自己的目标，即使读数回落超出建议的范围内。
  - ◊ 如果您在一天测量超过一次，这只能计算为一次测量。
  - ◊ 如果您在连续的一天测量，这一天将不会计入您的目标。例如，如果您周三测量，周四的测量将不计入您的目标。

- 每日收到0.50新元要是您照建议或您的eCAP记录服用药物。
  - ◊ 为了要被算进自己的目标，您将需要在适当的进餐时间服用您的糖尿病药物。

- 每日1.00新元，要是您的Fitbit Zip记录显示您有步行8,000步。

- 如果您达到您的目标，您可以收到价值高达每周14新元的奖励。

数据计算和奖励付款方式

- 研究分支联络人将通过检查您的研究设备的数据计算奖励（例如：**Fitbit**计步器、电子容器**eCAP**、和血糖仪）

- 在您的**第 3 和 6 个月评估**时，请携带全三样研究器材（**Fitbit**计步器、电子容器**eCAP**、和血糖仪）。

- 所有款项将在您的第3个月和6个月评估以**NTUC礼券**付给您（或者在下一次访问如果数据不能在访问期间计算完成）.

- **付款只会在您提供研究设备的数据后进行。**

附加信息

- 您的第一次监测评估期将从您的基线访问后的一天开始。这一监测期将是84天。您的奖励将以这84天的时间计算。
- 您的第二次监测评估期将从您第3个月访问后的一天开始。该监控期也将持续84天。您的奖励将以这84天的时间计算。
- 您会因参加这项研究，完成第三个月和第六个月的评估而各获得$15的礼券。
- 研究完成过后，您必须退还您拿到的Fitbit和eCAP.
- 如果参加者没有自己的血糖测计仪，那他在研究开始时收到的血糖测计仪将能在研究过后留着继续使用。
