# Supplementary material for: Trial to Incentivise Adherence for Diabetes (TRIAD): study protocol for a randomised controlled trial
Source: Trials. 2017 Nov 17;18:551. doi: 10.1186/s13063-017-2288-6 (PMC5693491; doi:10.1186/s13063-017-2288-6)
Supplement: Supplementary file 6 — Excerpt of TRIAD Participant Instruction Booklet (Outcome Incentive – English). This booklet is given to all English-speaking Outcome Incentive arm participants when they join the study. The booklet reminds the participants of the diabetes management recommendations, gives detailed explanations of the goals and incentives for this study arm, and provides information on the use of the study devices. (PDF 227 kb) [file 13063_2017_2288_MOESM6_ESM.pdf]

Recommendations to optimally manage your diabetes

- Aim to have blood sugar levels **between 4.0 and 7.0mmol/L** before your meals.
- Measure your blood sugar levels at least on **3 non-consecutive days** in the week.
- The best time to test your blood sugar is before you take your breakfast.
- You are also recommended to test your blood sugar 2 hours after your meals.
    - readings should be below 8.5 mmol/L if you have type 2 diabetes.
- Aim to take **8,000 steps** every day.
- Take your diabetes medication(s) as recommended each day.
- The Site Study Coordinator will note in the table below when you should take your medication:

Name of diabetes medication	Breakfast	Lunch	Dinner

Goals and financial incentives

Record healthy blood sugar readings

You will earn incentives for recording glucose readings **between 4 to 7mmols/L** **before a meal** on **3 non-consecutive days** within the week using your glucometer:

- SGD **2** weekly if one of your glucose readings falls within the normal range,

or:

- SGD **7** weekly if two of your glucose readings fall within the normal range,

or:

- SGD **14** weekly if all three of your glucose readings fall within the normal range

If you meet your goals you can receive incentives worth up to SGD **14** per week.

Calculations and payment of financial incentives

- In case you test more than once during a day, only the first reading will count towards your goal.
- In case you test on a consecutive day, the corresponding reading(s) will not count towards your goal. For instance, if you test on Wednesday, all readings recorded on Thursday will not count towards your goal.
- Only **the first three non-consecutive days of testing** of the week will be counted. For instance, if you test on Monday, Wednesday and Friday, all readings recorded on Saturday and Sunday will not count towards your goal.
- The Site Study Coordinator will calculate the incentives by checking your data on your glucometer.
- All payments will be made in the form of NTUC vouchers at your month 3 and 6 assessments (or at the next visit if the incentive cannot be computed during the visit).
- <u>Payment will only be made if data recorded by your glucometer is provided</u>.

Additional information

- Your first monitoring assessment period will start the day after your Baseline visit. This monitoring period will be for 84 days. Your incentives will be calculated based on this 84 day period.

- Your second monitoring assessment period will start the day after your month 3 visit. This monitoring period will also last 84 days. Your incentives will be calculated based on this 84 day period.
- You will receive $15 when you join the study, $15 for completing the Month 3 Visit, and $15 for completing the Month 6 Visit.
- Please bring the 3 study devices (Fitbit, eCAP and glucometer) to your Month 3 and Month 6 assessments.

- You will be required to return the Fitbit and eCAP at the end of the study.
- Participants who do not have their own glucometer and are given a glucometer at the beginning of the study may keep the glucometer after the study has finished if they wish.
