# Supplementary material for: Trial to Incentivise Adherence for Diabetes (TRIAD): study protocol for a randomised controlled trial
Source: Trials. 2017 Nov 17;18:551. doi: 10.1186/s13063-017-2288-6 (PMC5693491; doi:10.1186/s13063-017-2288-6)
Supplement: Supplementary file 7 — Excerpt of TRIAD Participant Instruction Booklet (Outcome Incentive – Mandarin). This booklet is given to all Mandarin-speaking Outcome Incentive arm participants when they join the study. The booklet reminds the participants of the diabetes management recommendations, gives detailed explanations of the goals and incentives for this study arm, and provides information on the use of the study devices. (PDF 351 kb) [file 13063_2017_2288_MOESM7_ESM.pdf]

最适合治疗您的糖尿病的建议

- 目标是在吃饭前，血糖值应该要在4.0 至 7.0 mmol/L之间。
- 一个星期里在**三个非连续天**测量您的血糖值。
- 最好测量血糖值的时间是在吃早餐前。
- 我们也建议您吃饭后两个小时，测量您的血糖值。

◊您的血糖应该在8.5mmol/L 以下要是您有2型糖尿病

- 目标是每天走8,000步。
- 服用每天所推荐的糖尿病药物。
- 研究分支联络人会在研究以下的表写您应该几时吃您的药物:

糖尿病药物的名称	早餐	午餐	晚餐

目标和金钱奖励

记录健康的血糖读数

您将获得奖励要是您使用您的血糖仪在一周之内3个非连续天用餐前的血糖读数是4至7mmols/ L之间：

- 每周2新元，如果您的一个血糖读数是在正常范围之内，

或：

- 每周7新元，如果您的两个血糖读数是在正常范围之内，

或：

- 每周14新元，如果您所有三个血糖读数是在正常范围内。

- 如果您达到您的目标，您可以收到价值高达每周14新元的奖励。

请注意下列条件：

- 如果您在一天测试多次，只有第一个读数将计入您的目标。
- 如果您连续两天测试，相应的读数将不计入您的目标。例如，如果您在周三测试，周四所有读数将不计入您的目标。
- 一周的测试中，只有前三个不连续的日子将被计算在内。例如，如果您在周一，周三和周五测试，周六和周日所有读数将不计入您的目标。
- 研究分支联络人将通过检查您的血糖仪的数据计算奖励。
- 所有款项将在您的第3个月和6个月评估以**NTUC礼券**付给您（或者在下一次访问如果数据不能在访问期间计算完成）。
- 付款只会在您提供研究设备的数据后进行。

附加信息

- 您的第一次监测评估期将从您的基线访问后的一天开始。这一监测期将是84天。您的奖励将以这84天的时间计算。

- 您的第二次监测评估期将从您第3个月访问后的一天开始。该监控期也将持续84天。您的奖励将以这84天的时间计算。

- 您会因参加这项研究，完成第三个月和第六个月的评估而各获得$15的礼券。

- 在您的**第 3 和 6 个月评估**时，请携带全三样研究器材（Fitbit计步器、电子容器eCAP、和血糖仪）。

- 研究完成过后，您必须退还您拿到的Fitbit和eCAP。

- 如果参加者没有自己的血糖测计仪，那他在研究开始时收到的血糖测计仪将能在研究过后留着继续使用。
