# Supplementary material for: Trial to Incentivise Adherence for Diabetes (TRIAD): study protocol for a randomised controlled trial
Source: Trials. 2017 Nov 17;18:551. doi: 10.1186/s13063-017-2288-6 (PMC5693491; doi:10.1186/s13063-017-2288-6)
Supplement: Supplementary file 8 — Participant Information Sheet and Consent Form (English). All English-speaking participants will complete this Consent Form before joining the study. The participant signs two copies, one copy is kept by SingHealth Polyclinics and one copy is given to the participant. (PDF 438 kb) [file 13063_2017_2288_MOESM8_ESM.pdf]

PARTICIPANT INFORMATION SHEET

You are being invited to participate in a research study.

Before you take part in this research study, the study must be explained to you and you must be given the chance to ask questions. Please read carefully the information provided here. If you agree to participate, please sign the informed consent form. You will be given a copy of this document to take home with you.

STUDY INFORMATION

Protocol Title:

A Randomised Controlled Trial to Improve Diabetes Outcomes through Financial Incentives

Simplified Title: Trial to Incentivise Adherence for Diabetes (TRIAD)

Principal Investigator:

Dr. Marcel Bilger

Health Services and Systems Research

Duke-NUS Graduate Medical School

8 College Road, Singapore (169857)

Tel: (65) 6601 2330

Site Principal Investigator:

Dr. Shah Mitesh

SingHealth Polyclinics – Geylang Polyclinic

21 Geylang East Central Singapore 389707

Tel: (65) 6547 6942

Sponsor:

NUS Global Asia Institute

PURPOSE OF THE RESEARCH STUDY

You are being invited to participate in a research study of financial incentives and medical adherence among diabetic patients. Medical adherence refers to how much patient behaviour matches what is recommended by healthcare professionals. We hope to learn about the effectiveness of financial incentives on encouraging medical adherence.

You were selected as a possible subject in this study because:

- your diabetes control is sub-optimal (i.e. have at least 1 of 2 HbA1C readings of 8.0 or greater in the past 6 months) and
- you have been on diabetic medication (not insulin) for at least 3 months .

This study will recruit 240 participants from Geylang Polyclinic over a period of 2.5 years.

Note that the HbA1C blood test will be performed on a portable kit at the polyclinic's laboratory and no blood sample will be stored for the purpose of this study. You will not have to pay for these blood tests.

STUDY PROCEDURES AND VISIT SCHEDULE

As part of this study, participants will each be given a Fitbit Zip (an electronic step-activity tracker) and an eCAP (an electronic container cap). When your participation in the study ends, you will no longer have access to the Fitbit Zips or eCaps and you will need to return these devices to the Site Study Coordinator. In addition, participants who do not own a supported glucometer (to measure blood sugar levels) will be given one at the beginning of the study. If you are given a glucometer, you will be offered to keep the device when the study ends.

If you agree to take part in this study, you will be randomised to 1 of 3 study groups. Randomisation means putting you in one of 3 groups by chance, like tossing a coin or rolling a dice. Neither the participant nor study staff will be able to decide or change group allocation drawn.

Group 1 Control Group: Diabetes Educational Program (DEP)

Participants in this group will receive the DEP which is delivered by a Health Counsellor at the point of diagnosis and focused education is provided during doctor visits for medication. At the end of the study, a fairness payment of SGD 75 in NTUC vouchers is given to all participants who fulfil all study requirements.

Group 2 Process Incentive Group: DEP and Incentives for Process Adherence

Participants in this group will receive an incentive scheme in addition to the DEP. Incentives will be paid in the form of NTUC vouchers and can be earned if participants meet these goals:

- SGD **3.50** <u>weekly</u> for measuring their blood sugar levels on 3 non-consecutive days in the week. This will be tracked using the glucometer.
- SGD **0.50** <u>daily</u> for taking medications as recommended each day. This will be tracked using the eCAP.
- SGD **1.00** <u>daily</u> for taking 8,000 steps every day. This will be tracked by the Fitbit Zip.

In total, participants can receive incentives worth up to SGD 14 per week. This will be done by checking stored data on the study devices (i.e. Fitbit, eCAP and glucometer).

All payments will be made in the form of NTUC vouchers at the 3- and 6-month visits only if data recorded by study devices is provided and the participant complete study procedures.

Group 3 Outcome Incentive Group: DEP and Incentives for Outcome Adherence

Participants in this group will receive the DEP and an incentive scheme similar to that offered in Group 2.

Participants will earn incentives for recording glucose readings within the normal range (i.e. between 4 to 7mmols/L before a meal) on **3 non-consecutive days** within the week using the glucometer:

- SGD **2** weekly if one glucose readings falls within the normal range,

or:

- SGD **7** weekly if two glucose readings fall within the normal range,

or:

- SGD **14** weekly if all three glucose readings fall within the normal range

If participants meet the goals they can receive incentives worth up to SGD **14** per week.

Incentive payments will be made in the form of NTUC vouchers to participants at Months 3 and 6 after glucometer readings are checked.

Participants in all 3 study groups will receive SGD15 when they join the study, SGD15 for completing the Month 3 Visit and SGD15 for completing the Month 6 Visit.

Schedule of visits and procedures:

If you agree to take part in this study, you will be asked to attend at least 3 study visits 1 at Baseline (i.e. when study starts) and 2 follow-ups at 3 and 6 Months. Your participation in the study will last at least 6 months.

Information on your medical record or pharmacy claims relating to your diabetic condition such as the number of diabetic medications, dose frequency and number of prescription refills will also be retrieved and used for analyses. All such information will be de-identified at the earliest opportunity and will only be used by the study team for the purpose of this study.

	**Questionnaire**	**Study devices issued / data downloaded**	**Blood test (HbA1C)**	**Incentives paid* (if applicable)**
**Baseline visit**	✓	✓	✓	✓
**Month 3 follow-up visit**	X	✓	X	✓
**Month 6 follow-up visit**	✓	✓	✓	✓

***In the unlikely event that incentives cannot be calculated during the clinic visit, payments will be made during the next scheduled clinic visit.**

Additional Study Procedures

Participants without a Personal Computer or smartphone will be asked to meet with study

staff once each month briefly (i.e. for 5 minutes) so activity data can be retrieved from their Fitbit Zip device.

Additional Blood Tests

Participants may test their blood sugar levels while participating in this study. To do so, a small drop of blood may be required each time. Also, a blood sample will be required to test for HbA1c levels at Baseline, as well as at the Month 6 visit. Participants will not have to pay for these blood tests.

YOUR RESPONSIBILITIES IN THIS STUDY

If you agree to participate in this study, you should:

- Use the study devices as instructed and follow the advice given to you by the study team.
  - The Fitbit Zip should be worn daily and ideally at the same location each time it is worn.
  - The eCAP should only be opened when you take your medicine(s), and shut thereafter.
  - The lancet used with Glucometer should always be disposed of after use.
- Be prepared to visit Geylang Polyclinic at least 3 times and undergo all the procedures that are outlined above. Care will be taken to coordinate study visits with scheduled clinic visits to minimize participation burden.
- Keep your study appointments. If it is necessary to miss an appointment, please contact the Site Study Coordinator or Site Principal Investigator to reschedule as soon as you know you will miss the appointment.
- Inform your attending doctor/the Site Study Coordinator as soon as possible about any side effects that you may have encountered.

WITHDRAWAL FROM STUDY

You are free to withdraw your consent and discontinue your participation at any time without prejudice to you or effect on your medical care. If you decide to stop taking part in this study, you should inform the research staff and Principal Investigator.

If you withdraw from the study:

- Please contact the Site Study Coordinator or Site Principal Investigator.
- You will be asked to return all study devices (Fitbit, eCAP and the glucometer if you received one) to the study team. This should be arranged with the Site Study Coordinator.

Your doctor, the Site Principal Investigator and/or the Sponsor of this study may stop your participation in the study at any time for one or more of the following reasons:

- Failure to follow the instructions of the Site Study Coordinator or Site Principal Investigator.
- The Site Principal Investigator decides that continuing your participation could be harmful.
- Pregnancy.
- It is possible that, during the course of the 6-month intervention, the condition of a participant is deemed unsatisfactory by his/her doctor (typically during the Month 3 visit). At this point, the doctor may advise the individual to start using insulin,

which would mean the individual is no longer eligible for the study. If this decision is made based on blood sugar levels, the Site Study Coordinator takes note of the HbA1c level, proceeds with the Month 3 protocol but does not schedule a Month 6 follow-up visit. The Site Study Coordinator also proceeds with the payment of SGD 45 to the patient in compensation for forgoing potential payments that the patient might have received had s/he remained in the study. This compensation amounts to half the total incentives received by control group participants during the 6-month intervention.

- The study is cancelled.

WHAT IS NOT STANDARD CARE OR EXPERIMENTAL IN THIS STUDY

The study is being conducted because the use of financial incentives and study devices Fitbit and eCAP are not used as part of the usual care in subjects with diabetes who are not on insulin therapy. We hope that your participation will help us to determine whether the incentive treatment(s) is equal or superior to existing usual cares procedures. Please note that financial incentives are offered as part of the study, and will only be used throughout the study duration.

Although components of the Diabetes Educational Program (DEP) may be part of standard medical care, in this study this / these procedure(s) are being performed for the purposes of the research.

POSSIBLE RISKS, DISCOMFORTS AND INCONVENIENCES

The Diabetes Educational Program (DEP), which is offered to all participants, is adapted from the usual care offered to diabetic patients at Geylang Polyclinic and will be conducted by trained and qualified healthcare professionals. Additionally, the incentive schemes do not involve recommendations or activity beyond what is covered under the DEP. As such, this study is considered low risk and involves no greater risks than would occur for any individual on a typical care schedule. Fitbit use (if applicable) will pose minimal inconvenience or discomfort given its size and weight.

POTENTIAL BENEFITS

If you participate in this study you may reasonably expect to benefit from the study intervention. The DEP program will provide participants with the skills and knowledge to manage their diabetic condition through consultations or sessions with healthcare professionals. To the extent that you respond and adhere to the instructions given as part of this program, better management of and diabetic outcomes are expected. Additionally, your participation will also contribute to the medical knowledge regarding the use and cost-effectiveness of using financial incentives and tracking devices on medical adherence behaviour. You may also earn incentives for your participation.

SUBJECT'S RIGHTS

Your participation in this study is entirely voluntary. Your questions will be answered clearly and to your satisfaction.

In the event of any new information becoming available that may be relevant to your willingness to continue in this study, you or your legal representative will be informed in a timely manner by the Principal Investigator or his/her representative.

By signing and participating in the study, you do not waive any of your legal rights to revoke your consent and withdraw from the study at any time.

CONFIDENTIALITY OF STUDY AND MEDICAL RECORDS

Information collected for this study will be kept confidential. Your records, to the extent of the applicable laws and regulations, will not be made publicly available. Only your Investigator(s) will have access to the confidential information being collected.

However, the Sponsoring company *(NUS GAI)*, Regulatory Agencies, Institutional Review Board and Ministry of Health will be granted direct access to your original medical records to check study procedures and data, without making any of your information public.

By signing the Informed Consent Form attached, you or your legal representative are authorizing (i) collection, access to, use and storage of your "Personal Data, and (ii) disclosure to authorised service providers and relevant third parties.

"Personal Data" means data about you which makes you identifiable (i) from such data or (ii) from that data and other information which an organisation has or likely to have access. This includes medical conditions, medications, investigations and treatment history.

Research arising in the future, based on this Personal Data, will be subject to review by the relevant institutional review board.

By participating in this research study, you are confirming that you have read, understood and consent to the SingHealth Data Protection Policy- the full version is available at www.singhealth.com.sg/pdpa. Hard copies are also available on request.

Data collected and entered into the Data Collection Forms are the property of Duke-NUS Graduate Medical School. Only de-identified data will be collected by Duke-NUS Graduate Medical School. In the event of any publication regarding this study, your identity will remain confidential. Duke-NUS Graduate Medical School may request permission from Geylang Polyclinic to access your de-identified Hba1c (i.e. blood sugar levels) up to 2 years after you have completed the study.

COSTS OF PARTICIPATION

If you take part in this study, the following will be performed at no charge to you:

- Delivery of the Diabetes Education Program (DEP)
- A HbA1c blood test administered twice: at Baseline and Month 6

  At your request, if you have had the HbA1c test taken within 1 week of enrolment, the results from that test can be used and re-testing would not be required for the Baseline visit.

  Note: The study will cover the cost for 2 HbA1c blood tests for each participant. For participants who have been tested within 1 week of recruitment and have already paid for the test, the Site Study Coordinator will arrange for reimbursement through the Polyclinic.

If you take part in this study, you will have to pay for the following:

- Costs incurred as part of your usual care schedule such as for Doctor Consultations, Diabetes Medication, and Test strips or lancets for glucose/glucometer testing.

You will be reimbursed for your time, inconvenience and transportation costs as follows:

Condition	Incentive	Group allocation: 1 of 3 groups		
		1. Control	2. Process	3. Outcome
Meeting specified process OR outcome goals	Varies (SGD0 to SGD336)		✓	✓
Fairness payment (for control group participants who provided all data and attended all visits)	SGD75	✓		
For all participants recruited to the study (at Baseline assessment)	SGD15	✓	✓	✓
For participants who provided all data and attended the Month 3 visit	SGD15	✓	✓	✓
For participants who provided all data and attended the Month 6 visit	SGD15	✓	✓	✓
Total earned incentives: NTUC vouchers worth		SGD120	, SGD45 to SGD381, depending on met goals	SGD45 to SGD381, depending on met goals

Payments will be made in the form of NTUC vouchers at study visits. If you do not complete the study for any reason, you will still receive all earned incentives based on the data you provide. You will receive an additional SGD45 in compensation if your doctor or a principal investigator decides to stop your participation in this study.

RESEARCH RELATED INJURY AND COMPENSATION

The Polyclinic does not make any provisions to compensate study subjects for research related injury. However, compensation may be considered on a case-by-case basis for unexpected injuries due to non-negligent causes.

By signing this consent form, you will not waive any of your legal rights or release the parties involved in this study from liability for negligence.

WHO TO CONTACT IF YOU HAVE QUESTIONS

If you have questions about this research study or in the case of any injuries during the course of this study, you may contact the Site Study Co-ordinator Hui Yan Xu at 98383446,

or the Site Principal Investigator Dr. Shah Mitesh at (65) 6547 6942.

This study has been reviewed by the SingHealth Centralised Institutional Review Board for ethics approval.

If you have questions about your rights as a participant, you can call the SingHealth Centralised Institutional Review Board at 6323 7515 during office hours (8:30 am to 5:30pm).

If you have any complaints about this research study, you may contact the Principal Investigator or the SingHealth Centralised Institutional Review Board.

CONSENT BY RESEARCH SUBJECT	
**Details of Research Study**	
**Protocol Title:**<br>A Randomised Controlled Trial to Improve Diabetes Outcomes through Financial Incentives (TRIAD)<br>**Principal Investigator:** Dr. Marcel Bilger, Health Services and Systems Research, Duke-NUS Graduate Medical School, 8 College Road, Singapore (169857) Tel: (65) 6601 2330.<br>**Site Principal Investigator:** Dr. Shah Mitesh, SingHealth Polyclinics – Geylang Polyclinic, 21 Geylang East Central Singapore 389707 Tel: (65) 6547 6942	
**Subject's Particulars**	
Name:<br>Address:	NRIC No.:
Sex: Female/Male	Date of birth ______________<br>dd/mm/yyyy
Race: Chinese/ Malay/ Indian /Others (please specify) ______________________	

I, ___________________________________(NRIC/Passport No.______________________)
(Name of patient)
agree to participate in the research study as described and on the terms set out in the Patient Information Sheet.

I have fully discussed and understood the purpose and procedures of this study. I have been given the Participant Information Sheet and the opportunity to ask questions about this study and have received satisfactory answers and information.

I understand that my participation is voluntary and that I am free to withdraw at any time, without giving any reasons and without my medical care being affected.

By participating in this research study, I confirm that I have read, understood and consent to the SingHealth Data Protection Policy. I also consent to the use of my Personal Data for the purposes of engaging in related research arising in the future.

_____________________________________ _____________________
Signature/Thumbprint (Right / Left) of patient Date of signing

To be filled by parent / legal guardian / legal representative, where applicable

I, ____________________________ hereby give consent for the above participant to participate in
(parent / legal guardian)
the proposed research study. The nature, risks and benefits of the study have been explained clearly to me and I fully understand them.

____ ____________________________
Signature/Thumbprint (Right / Left) of parent /legal guardian Date of signing

Translator Information (if required)

The study has been explained to the participant/ legal representative in

_____________________________________by ______________________________________.
Language Name of translator

To be filled witness, where applicable

An impartial witness should be present during the entire informed consent discussion if a subject or the subject's legal representative is unable to read. After the written informed consent form and any written information to be provided to subjects, is read and explained to the subject or the subject's legal representative, and after the subject or the subject's legal representative has orally consented to the subject's participation in the study and, if capable of doing so, has signed and personally dated the consent form, the witness should sign and personally date the consent form.

Witnessed by: _________________________________ ____________________________
Name of witness Designation of witness

_________________________________ ____________________________
Signature of witness Date of signing

Investigator's Statement

I, the undersigned, certify to the best of my knowledge that the patient/patient's legal representative signing this informed consent form had the study fully explained and clearly understands the nature, risks and benefits of his/her / his ward's / her ward's participation in the study.

_________________________ _________________________ ________________
Name of Investigator/ Signature Date
Site Study Coordinator
