# Supplementary material for: Trial to Incentivise Adherence for Diabetes (TRIAD): study protocol for a randomised controlled trial
Source: Trials. 2017 Nov 17;18:551. doi: 10.1186/s13063-017-2288-6 (PMC5693491; doi:10.1186/s13063-017-2288-6)
Supplement: Supplementary file 9 — Participant Information Sheet and Consent Form (Mandarin). All Mandarin-speaking participants will complete this Consent Form before joining the study. The participant signs two copies, one copy is kept by SingHealth Polyclinics and one copy is given to the participant. (PDF 562 kb) [file 13063_2017_2288_MOESM9_ESM.pdf]

参与者信息表

在此，您被邀请参与一项研究。

在您决定是否参与本研究前， 我们将向您详细介绍本研究，并且将给予您提出疑问的机会。请认真阅读这份资料。若您愿意参与本研究, 请您在知情同意书上签字。您将会得到一份此文件。

研究资料

研究名称:

一个随机对照试验，以通过金钱激励提高糖尿病结果

简化标题: 奖励糖尿病人坚持用药的试验 (TRIAD)

研究总负责人:

Marcel Bilger 博士

卫生系统与服务研究部

杜克-新加坡国立大学医学研究生院

地址: 8 College Road Singapore 169857

电话: (65) 6516 2338

研究分支总负责人

Shah Mitesh 医生

新加坡保健集团综合诊所– 芽笼综合诊所

地址：21 Geylang East Central Singapore 389707

电话：(65) 6547 6942

资助机构:

新加坡国立大学亚洲国际学院

研究目的

你被邀请参加金钱奖励和糖尿病患者的医疗坚持的一项研究。医疗坚持是指患者的行为有多符合由专业医护人员的建议。我们希望了解金钱奖励鼓励医疗坚持的有效性。

您被选为这项研究的可能参与者，因为：

- 你的糖尿病控制是次优（即在过去6个月至少有二分之一HbA1C读数大于或等于8）和
- 您为至少3个月一直用糖尿病药物（非胰岛素）。

这项研究将历时2.5年从芽笼综合诊所招募240参与者。

需要注意的是HbA1C的血液测试将用便携式药盒在综合诊所的实验室中进行，并没有血液样本将被储存作为这项研究。你将不必支付这些血液测试。

研究程序及检查时间表

作为这项研究的一部分，参加者将分别给予一个 Fitbit Zip（电子计步器）和一个 eCAP（电子容器盖）。当您参与的这项研究结束后，你将不再获得 Fitbit Zip 或 eCAPs，你需要交回这些设备给研究分支联络人。此外，如果参与者没有自己的血糖仪（测血糖水平）他将在研究开始时获得一个。如果获得一个血糖仪，你可以在研究结束时保留它。

如果您同意参加这项研究，你会被随机分配到三个之一的研究小组。随机化是把你在三组中随机放在其中一组，像掷硬币或滚动骰子。小组分配的绘制是无论参与者或研究人员所能决定或改变的。

第 1 组对照组：糖尿病教育项目 (DEP)

在本组的参与者将收到这是由卫生参赞发表在诊断点和重点的教育过程中的用药医生门诊提供的 DEP。在研究结束时，一个 75 新元 NTUC 券的公平补贴是给谁满足所有要求的研究参与者。

第 2 组 过程奖励组：DEP 和过程中坚持的奖励

在本组的参与者将获得除了 DEP 之外的奖励计划。如果参与者达到这些目标，奖励将以 NTUC 券的形式支付：

- <u>每周</u> 3.50 新元，要是您在一周之内 3 个非连续天使用您的血糖仪测量您的血糖值。
- <u>每日</u>收到 0.50 新元要是您照建议或你的 eCAP 记录服用药物。
- <u>每日</u> 1.00 新元，要是你的 Fitbit Zip 记录您有步行 8,000 步。

总体而言，参与者可获赠价值高达每周 14 新元的奖励。这将通过检查学习设备（如 Fitbit，ECAP 和血糖仪），存储的数据来完成。

所有款项将会在 NTUC 券在 3 和 6 个月的访问形式只有通过记录设备的研究数据提供和参与者完整的研究过程。

第 3 组 结果奖励组：DEP 和成果坚持的奖励

在本组的参与者将获得 DEP 和类似提供给 2 组的奖励计划。

如果在一周内的 **3 个非连续天**，使用血糖仪所记录的血糖读数在正常范围内（饭前血糖值即在 4 至 7mmols/ L 之间）参与者将获得奖励：

- 每周 2 新元，如果您的一个血糖读数是在正常范围之内，

或：

- 每周 7 新元，如果您的两个血糖读数是在正常范围之内，

或：

- 每周 14 新元，如果您所有三个血糖读数是在正常范围内。

如果您达到您的目标，您可以收到价值高达每周 14 新元的奖励。

奖励金将会以 NTUC 礼券的形式，在 3 个月和 6 个血糖仪读数进行检查后支付。

无论您进哪个研究组，研究参与者都会在因参加本研究，完成第三个月和第六个月的研究评估后而各获得 15 新元。

访问日程安排和程序：

如果您同意参加这项研究，你会被要求参加至少 3 个考察访问 1 个在基线（即研究开始时）和 2 个跟进访问，在 3 和 6 个月。您参与这项研究将持续至少 6 个月。

有关你的糖尿病病情的病历或药房索赔，如多少种糖尿病药物，剂量频率和处方数量的信息也将被回收并用于分析。所有这些信息将被尽早取消认定并只让研究小组作为研究用途。

	问卷	研究设备发出/下载数据	验血 (HbA1C)	支付奖励*（如果适用）
基线访问	✓	✓	✓	✓
3 个月随访	X	✓	X	✓
6 个月随访	✓	✓	✓	✓

***万一奖励不能在门诊访问期间计算，款项将在下一个计划访问的期间支付。**

额外的研究过程

没有个人电脑或智能手机的参与者将被要求和研究人员每月一次短暂会面（即 5 分钟），以便活动数据可以从他们的 Fitbit Zip 设备进行检索。

额外的血液测试

参与者可以在参加这项研究的同时测试他们的血糖水平。要做到这一点，每一次可能需要一小滴血。此外，在基线以及在 6 月访问血液样本会被用来测试 HbA1c 水平，与会者将不必支付这些血液测试。

您在本研究中的责任

如果您同意参与这项研究，您应该：

- 依照指示使用研究设备，并按照研究团队给你的建议。
  - Fitbit Zip 应当每天和最好每次在相同的位置佩戴。
  - eCAP 应该只有在你取用你的药物时打开，然后关掉。
  - 和血糖仪一起使用的刺血针应当在使用后丢弃。

- 准备好访问芽笼综合诊所至少 3 次，并经过所有上面列出的步骤。将小心协调研究访问和已安排就诊访问，以尽量减少参与的负担。
- 坚持你的研究预约。如果有必要错过预约，在你知道你会错过预约时，请联系研究分支联络人或研究分支总负责人尽快重新安排。
- 尽快通知您的主治医生/研究分支联络人任何你可能遇到的副作用。

退出研究

在不对您引起偏见，不影响您的医疗服务的情况下，您可在研究过程中随时撤消同意并退出研究。若您决定退出研究, 请您通知研究人员和研究总负责人。

若您退出研究:

- 请通知研究分支联络人或研究分支总负责人。
- 你会被要求退还所有研究设备(Fitbit、电子容器（eCAPs）和血糖仪如果您收到一个)给研究小组。请您和研究分支联络人安排。

您的医生、研究总负责人及/或研究赞助商，有权因以下一或多种原因，在任何时间中止您在本研究中的参与：

- 您未能遵循研究分支联络人或研究分支总负责人的指令。
- 研究分支总负责人认为继续让您参与可能带来害处。
- 怀孕。
- 在 6 个月的介入的过程中，一个参与者的状况（通常在第 3 个月的访问）有可能不能让他/她的医生感到满意。这时候，医生可能会告知参与者开始使用胰岛素。在这种情况下，患者就不再有资格参加研究。如果根据血糖水平作出这个决定，研究分支联络人会注意到 HbA1c 水平，继续进行第 3 个月的协议，但不安排 6 个月后续访问。研究护士会与支付 45 新元给患者做放弃的赔偿金，等于患者未来可能收到的付款，如果他继续参加研究。这个补偿金等于对照组的参与者在 6 个月介入中收到的一半的总奖励。
- 本研究被中断。

在这项研究中什么是不标准治疗或实验

这项研究正在开展，因为使用金钱奖励和研究设备 Fitbit 和 eCAP 不作为对没有使用胰岛素治疗的糖尿病患者常规看护的一部分。我们希望您的参与将帮助我们确定激励处理是否等于或优于现有平时看护程序。请注意，金钱奖励被提供作为研究的一部分，并且将只在整个研究持续时间使用。

虽然糖尿病教育计划（DEP）的成分可以是标准医疗保健的一部份，在此研究中这些程序会被用做研究的目的。

可能产生之风险，不舒适和不便之处:

提供给所有的参与者的糖尿病教育计划（DEP），是改编自芽笼综合诊疗所提供给糖尿病患者的平时照顾，并会由经过培训和合格的医疗专业人士进行。此外，奖励计划不涉及在 DEP 所覆盖之外的建议或活动。因此，这项研究中被认为是低风险的，并且对会发生在一个使用典型的保健日程的人没有更大的风险。鉴于其尺寸和重量，Fitbit（如果适用）将带来最少的不便和

不适。

预期效益

如果您参加这项研究，你可以合理预期从研究干预中受益。该 DEP 课程将提供学员的技能和知识，通过与医疗专业人士进行协商或会话来管理他们的糖尿病病情。你对该计划部份说明的回应和坚持预计能更好的管理糖尿病的结果。此外，您的参与也将有助于有关使用金钱奖励和跟踪器对医药坚持行为的成本效益的医学知识。您也可以赚取您的参与奖励。

参与者权益

您参与这项研究是完全自愿的。您的问题将明确回答让您满意。

在得到任何新的、可能是有关您愿意在这项研究中继续的信息时，首席研究员或他/她的代表将既时通知您或您的法定代表人。

通过签署和参加研究，你不会放弃您的任何合法权利撤销许可，并在任何时候退出研究。

研究和医疗记录的保密性

收集作这项研究的信息将被保密。您的记录，在适用的法律和法规的范围内，不会被公开。只有你的研究员将有机会获得被收集的机密信息。

但是，赞助公司（*NUS GAI*）、监管机构、机构审查委员会和卫生部将被授权直接访问您的原始病历，以检查研究程序和数据，而不把您的任何信息公开。

通过签署附加知情同意书，您或您的法律代表将授权（i）收集、获取、使用和储存你的“个人资料”，及（ii）披露给授权的服务提供商和相关第三方。

“个人数据”是指能识别您的关于您的数据（i）从这些数据 或（ii）从这些数据和一个机构已经或可能有机会获取的其它信息。这包括医疗条件、药物、调查和治疗历史。

在未来，在此基础上的个人数据所产生的研究，将会受到相关机构审查委员会审查。

通过参加这项研究，你确认你已阅读、理解并同意新加坡保健集团数据保护政策，此政策的完整版可在 www.singhealth.com.sg/pdpa 得到。硬拷贝也可应要求提供。

收集并进入数据收集表格的数据是杜克 - 新加坡国立大学医学研究生院的财产。只有去确定数据将由杜克 - 新加坡国立大学医学研究生院收集。如果有出版任何关于这项研究的刊物，您的身份将严格保密。在你已经完成了研究后，长达 2 年之间，杜克 - 新加坡国立大学医学研究生院可以请求芽笼综合诊所的许可访问您的去识别化 HbA1c（即血糖水平）

参与研究的费用

如果您参与本研究，下面将免费为您进行：

- 交付糖尿病教育计划（DEP）
- 提供 HbA1c 的验血两次：在基线和 6 个月

根据您的要求，如果你在报名后的 1 周内有做 HbA1c 测试，就可以使用测试结果，也不需要在基线访问时重新测试。

注：该研究会为每个参与者支付 2个HbA1c 的血液测试。 如果参与者在招聘的 1 周内已经测试了，并已经支付测试的费用，在第三个月的访问时，研究分支联络人将会透过综合诊疗所安排补偿。

如果您参与本研究，你得支付以下各项：

- 成本支出为你平时的护理日程的一部分，如医生咨询，糖尿病药物和作为葡萄糖/血糖仪测试的测试条或采血针。

您将报销你的时间，不便和运输费用如下：

条件	奖励	组分配：3 组中的 1 组		
		1. 对照	2. 过程	3. 结果
达到指定的进程或结果	不等（0 新元到 336 新元）		✓	✓
公平支付（给提供所有数据，并出席了所有访问的控制组的参与者）	75 新元	✓		
给所有在基线评估时参加本研究的参与者	15 新元	✓	✓	✓
给出席第三个月的研究评估，并提供所有数据的参与者	15 新元	✓	✓	✓
给出席第六个月的研究评估，并提供所有数据的参与者	15 新元	✓	✓	✓
共获得奖励：NTUC 券价值		120 新元	45 新元 到 381 新元,根据所满足目标	45 新元 到 381 新元,根据所满足目标

付款将以 NTUC 券的形式在考察访问时进行。如果以任何理由没有完成研究，你仍然会收到根据您所提供的资料的全部奖励。您将获得额外 45 新元的赔偿，如果你的医生或主要研究者决定停止你参加这项研究。

研究相关的损伤及赔偿

该综合诊所不作任何规定，以弥补研究对象进行研究有关的伤害。然而，补偿可考虑在逐案基础上对于因非疏忽导致意外伤害。

通过签署本同意书，你不会放弃你的任何法律权利或释放参与这项研究的当事人的疏忽责任。

你有问题时的相关联络人

如果您有问题有关此项研究或在这一研究过程中有任何伤害的情况下，您可以在<号码 TBC>联系研究分支联络人 Hui Yan Xu，或在（65）65476942 联系研究分支总负责人 Shah Mitesh 医生。

这项研究已通过新加坡保健机构集中审查委员会的审查和伦理批准。

如果您对您作为一个参与者的权利有问题，您可以于办公时间拨打新加坡保健集团集中机构审查委员会的电话 63237515（上午 8:30 至下午 5:30）。

如果您有任何关于这项研究的投诉，您可以联系首席研究员或新加坡保健集团集中机构审查委员会。

研究参与者同意书
**研究详情**
**研究名称:**
**一个随机对照试验，以通过金钱激励提高糖尿病结果(TRIAD)**
**研究总负责人：**Marcel Bilger 博士，卫生系统与服务研究部，杜克-新加坡国大医学研究生院，8 College Road，Singapore (169857) 电话：(65) 6601 2330.
**研究分支总负责人：**Shah Mitesh 医生, 新加坡保健集团综合诊所 –芽笼综合诊所, 21 Geylang East Central Singapore 389707 电话： (65) 6547 6942
**参与者个人资料**
名字: 身份证号码:<br>地址:<br><br>性别: 女/男 出生日期______________<br>日/月/年<br>种族: 华/巫/印/其他（请说明）: ______________________

我, ___________________________________(身份证/护照号码______________________)
(患者姓名)

同意依据参与者信息表所描述的条款参与以上的研究。

我已充分地讨论与了解本研究的程序及目的，也获得一份研究参与者信息表，有机会提出疑问,亦已得到满意的答案。

我了解我于本研究的参与是自愿性的，并可以在不需任何理由，在任何时间，在不影响我应有的医疗照顾的情况下自由地退出此研究。

参与本研究，我确认我已阅读、理解并同意接受新加坡保健集团资料保护政策。我也同意我的个人资料，使用于未来相关研究。

________________________________ ____________________

[参与者者签名/ 指印(右/左)] *(签名日期)*

患者家长/法定监护人/法定代理人，填写栏 （若有需要）

我 __________________________________ 同意上述参与者参与此研究计划。

（家长 / 监护人 ）

此研究的本质，风险以及好处已经清楚地向我解释和我清楚地明白。

__________________________________ ________________________

［家长/监护人签名/ 指印（ 右/ 左）］ （签名日期）

翻译者资料（若有需要）

已通过 ____________________用__________________向参与者或其法定代理

翻译者姓名 语言

人解释此研究的性质。

见证人填写栏（若有需要）

在患者或患者的法定代理人无法阅读的情况下，必须有一位公正的见证人在场见证整个签署和讨论知情同意书的过程。在书面同意书及任何书面资料已向患者或其法定代理人口头转述与解释之后，并且患者或其法定代理人已经口头同意参与本研究，及患者或其法定代理人有能力并已经亲自签署同意书并附上日期的情况下，见证人也必须亲自签署此栏并附上日期。

见证人：____________________ ______________________

（见证人名字） （见证人身份）

____________________ __________________

［见证人签名］ （签名日期）

研究负责人声明

我在此签名，并证明以我所知，这份同意书的签署人/签署人的法定代理人已经通过详尽的解释，清楚地了解他/她 /他/她的受监护人参加本研究的性质，风险和效益。

_________________________ ________________ ________________

研究负责人/ 签名 日期

研究分支联络人姓名
